# Supplementary material for: Assessment of Cd Pollution in Paddy Soil–Rice System in Silver Mining-Affected Areas: Pollution Status, Transformation and Health Risk Assessment
Source: Int J Environ Res Public Health. 2022 Sep 28;19(19):12362. doi: 10.3390/ijerph191912362 (PMC9564393; doi:10.3390/ijerph191912362)
Supplement: Supplementary file 1 [file ijerph-19-12362-s001.zip › ijerph-1899213-supplementary.pdf]

Supplementary materials

Table S1. Relationship between the BF and selected soil properties.

		pH	OM	Available K	Available P	CEC
BF	r	-0.305	-0.237	-0.064	-0.079	-0.231
	p	0.250	0.376	0.813	0.771	0.389

Table S2. Correlation between the Cd content in rice grain and various indicators.

	Cd in rice grain	
	r	p
Total Cd in soil	0.006	0.983
DTPA-extractable Cd	−0.219	0.414
$CaCl_2$-extractable Cd	−0.361	0.169
Acid-soluble Cd	−0.297	0.264
Reducible Cd	0.058	0.832
Oxidizable Cd	−0.280	0.293
Residual Cd	−0.080	0.768
pH Value	−0.149	0.583
OM	−0.293	0.271
Available K	−0.070	0.798
Available P	−0.037	0.893
CEC	−0.269	0.315

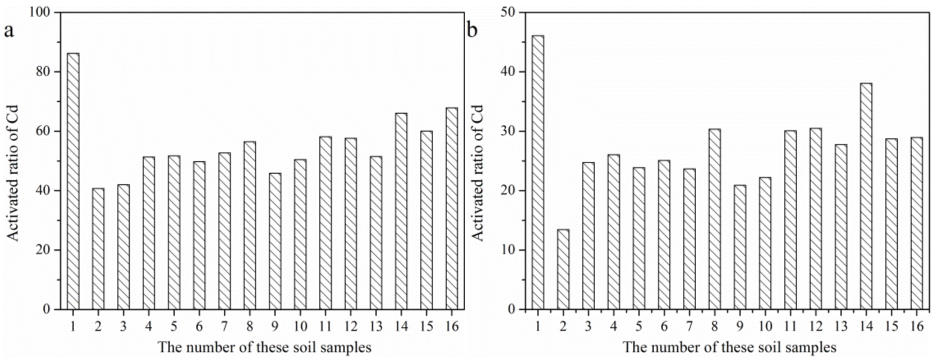

Figure S1. Activated ratio of Cd in these paddy soils, a: based on the extraction of DTPA; b: based on the extraction of $CaCl_2$.
